# Sodium sensing in the brain

**DOI:** 10.1007/s00424-014-1662-4

**Published:** 2014-12-10

**Authors:** Masaharu Noda, Takeshi Y. Hiyama

**Affiliations:** 1 Division of Molecular Neurobiology, National Institute for Basic Biology, 5-1 Higashiyama, Myodaiji-cho, Okazaki, 444-8787 Japan; 2 School of Life Science, The Graduate University for Advanced Studies, Okazaki, 444-8787 Japan

**Keywords:** Salt homeostasis, Na^+^ sensing, Na_x_ channel, Sensory circumventricular organs, Subfornical organ

## Abstract

Sodium (Na) homeostasis is crucial for life, and the Na^+^ level ([Na^+^]) of body fluids is strictly maintained at a range of 135–145 mM. However, the existence of a [Na^+^] sensor in the brain has long been controversial until Na_x_ was identified as the molecular entity of the sensor. This review provides an overview of the [Na^+^]-sensing mechanism in the brain for the regulation of salt intake by summarizing a series of our studies on Na_x_. Na_x_ is a Na channel expressed in the circumventricular organs (CVOs) in the brain. Among the CVOs, the subfornical organ (SFO) is the principal site for the control of salt intake behavior, where Na_x_ populates the cellular processes of astrocytes and ependymal cells enveloping neurons. A local expression of endothelin-3 in the SFO modulates the [Na^+^] sensitivity for Na_x_ activation, and thereby Na_x_ is likely to be activated in the physiological [Na^+^] range. Na_x_ stably interacts with Na^+^/K^+^-ATPase whereby Na^+^ influx via Na_x_ is coupled with activation of Na^+^/K^+^-ATPase associated with the consumption of ATP. The consequent activation of anaerobic glucose metabolism of Na_x_-positive glial cells upregulates the cellular release of lactate, and this lactate functions as a gliotransmitter to activate GABAergic neurons in the SFO. The GABAergic neurons presumably regulate hypothetic neurons involved in the control of salt intake behavior. Recently, a patient with essential hypernatremia caused by autoimmunity to Na_x_ was found. In this case, the hypernatremia was considered to be induced by the complement-mediated cell death in the CVOs, where Na_x_ specifically populates.

## Introduction

Terrestrial animals are exposed to considerable risks of dehydration and salt deficiency, and their life depends on the maintenance of water and salt in the body fluids [3, 4]. Changes in cell volume caused by severe hypertonicity or hypotonicity in body fluids can lead to irreversible damage to organs including nervous systems [5, 7, 62]. To escape from such risks, mammals have a set of homeostatic mechanisms that work together to maintain body fluid osmolality at approximately 300 mOsm/kg mainly through the intake or excretion of water and salt [37, 55]. When animals are dehydrated, both sodium ion concentration ([Na^+^]) and osmolality in body fluids increase because Na^+^ is the major cationic component of extracellular fluids and the main determinant of body fluid osmolality. Na^+^ homeostasis is thus inseparably linked with body fluid control, and [Na^+^] in body fluids needs to be continuously monitored to maintain its physiological range (135–145 mM for mammals) [4, 58].

Bengt Andersson hypothesized the existence of a putative [Na^+^] sensor within the brain nearly four decades ago that is distinct from osmosensors [3, 22]. Since then, a series of experimental evidences has suggested that the [Na^+^] increase in body fluids is detected in the brain [17, 48]. In addition, experimental ablation studies suggested that the anterior wall of the third ventricle is involved in [Na^+^] sensing [4, 17, 39]. However, the precise location of the [Na^+^]-sensing cells and the molecular entity of the [Na^+^]-specific sensor were not identified for a long time, though it has been a major interest of physiologists.

**Fig. 1:**
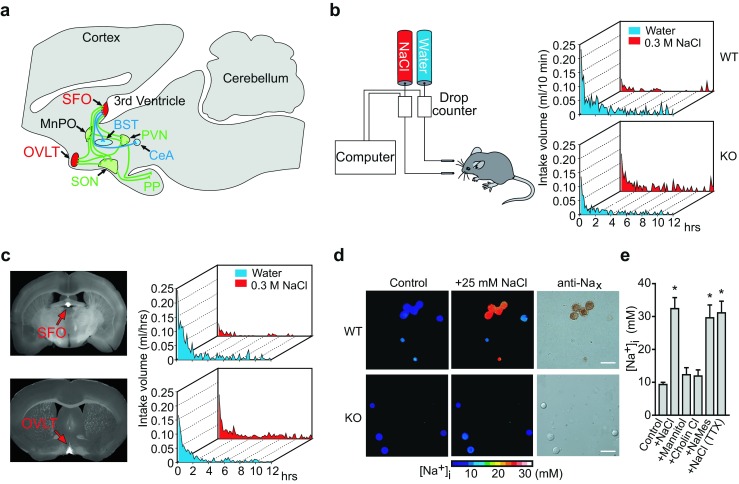
SFO is the primary locus of [Na^+^] sensing by Na_x_ channel for the control of salt intake behavior. **a** Neural connections for body fluid control in the brain. Na_x_-positive sensory circumventricular organs in the midsagittal section are schematically represented in *red*. Of note, Na_x_ is not expressed in the MnPO. *Green lines* indicate the neural connections involved in regulating the release of vasopressin; *blue lines* indicate putative neural connections involved in the control of water or salt intake. *SFO* subfornical organ, *MnPO* median preoptic area, *OVLT* organum vasculosum of the lamina terminalis, *BST* bed nucleus of the stria terminalis, *SON* supraoptic nucleus, *PVN* paraventricular nucleus, *CeA* central nucleus of the amygdala, *PP* posterior pituitary. **b** Averaged time course of water and saline (0.3 M NaCl) intake in wild-type (*WT*) and *Na*_*x*_-KO (*KO*) mice during the dark phase immediately after 48-h dehydration. Each *point* shows the averaged quantity per 10-min period of ten mice. **c** Coronal sections of mouse brains obtained from *Na*_*x*_-KO mice showing the infected loci by the expression of EGFP (*left column*). Time course of water and saline (0.3 M NaCl) intake by the infected mice after 48-h dehydration (*right column*). Behavioral data are the average of six mice that were successfully infected at a specific site in the brain by vectors encoding *Na*_*x*_ and *egfp*. All of the *Na*_*x*_-KO mice that were conferred with the Na aversion behavior showed a common infection in the SFO (*upper*). On the other hand, transduction of the Na_x_ gene into the OVLT could not rescue the abnormal salt intake behavior of the *Na*_*x*_-KO mice (*lower*). **d** Pseudocolor images showing the [Na^+^]_i_ of the cells in the control ([Na^+^]_o_ = 145 mM) and high-Na^+^ ([Na^+^]_o_ = 170 mM) solutions (*left and middle columns*) and immunocytochemical images using anti-Na_x_ antibody (*right column*). *Scale bars*, 50 μm. **e** The [Na^+^]_i_ response to various stimulations. The response was dependent on [Na^+^]_o_, but not on extracellular [Cl^−^]_o_ or osmotic pressure. Instead of 25 mM NaCl, 50 mM mannitol, 25 mM choline chloride (*Cholin Cl*), or 25 mM sodium methanesulfonate (*NaMes*) was added to the control solution. The response was not affected by 1 μM tetrodotoxin (*TTX*). *P < 0.001 by one-tailed Mann-Whitney tests. Reproduced with permission from [55] (**a**), [33] (**b**, **c**), and [34] (**d**, **e**)

The most likely candidate sites for [Na^+^] sensing were postulated to be the circumventricular organs (CVOs) of the brain [16, 17, 57]. The CVOs, midline structures found in the brain of all vertebrates, are so named because of their proximity to the ventricles of the brain [49]. Their specialized common features are extensive vascularization, no blood-brain barrier (BBB), and atypical ependymal cells being exposed to cerebrospinal fluid (CSF). Among the CVOs, only three loci, the subfornical organ (SFO), organum vasculosum of the lamina terminalis (OVLT), and area postrema (AP), harbor neuronal cell bodies that have efferent neural connections to many other areas of the brain. Therefore, these three CVOs are termed sensory CVOs [38] (Fig. 1a). Because of the lack of a BBB, their component cells are exposed to the chemical environment of the systemic circulation, unlike other neural cells in the central nervous system (CNS). The SFO and OVLT situated on the anterior wall of the third ventricle are supposed to be involved in the sensing of [Na^+^] and osmolality .

The cell type of [Na^+^]-sensing cells has also been a concern whether they are neurons or glial cells. It has been long considered that neurons transmit neural information and glial cells provide nourishment and physical scaffold for neurons. However, it is now widely accepted that glial cells can actively modulate the information processing by neurons via several mechanisms [28, 63].

## The primary locus of [Na^+^] sensing and the molecular entity of the [Na^+^] sensor

We have long been studying the functional roles of an atypical Na channel Na_x_, which was initially classified as a subfamily of voltage-gated Na channels and called Na_v_2 [51, 53, 54]. Na_x_ was originally cloned by several independent groups from rat astrocytes [23], the human heart [24], a mouse atrial tumor cell line [21], and the rat dorsal root ganglia (DRG) [2]. The primary structure of Na_x_ markedly differed from that of the other Na_v_ channel members, including the key regions for voltage sensing and inactivation [25, 51]. Because previous attempts at the functional expression of Na_x_ in heterologous systems had failed, we generated *Na*_*x*_-gene-knockout (*Na*_*x*_-KO) mice by inserting the *lacZ* gene in frame to examine the distribution and physiological roles of this channel [51, 65]. Na_x_ was revealed to be expressed in some limited loci in the brain, including the SFO and OVLT [65]. Na_x_ expression in these loci was confirmed by immunohistochemistry [67].

As the SFO and OVLT were the potential loci for [Na^+^] sensing, we examined the salt intake behaviors of the *Na*_*x*_-KO mice using the two-bottle test, providing them with both distilled water and a 0.3 M NaCl solution to drink [33, 65]. As long as wild-type (WT) and *Na*_*x*_-KO mice were fully satiated with water, they showed no marked preference for either [33]. However, when they were dehydrated, WT mice showed extensive water intake and aversion to saline, while *Na*_*x*_-KO mice did not show such an aversion to the saline (Fig. 1b) [33].

Because *Na*_*x*_-KO mice have a normal tasting ability, including that for salt, the behavioral defects in the *Na*_*x*_-KO mice were supposed to be attributable to some internal sensing mechanisms for [Na^+^] in body fluids [65]. Consistent with this view, infusion of a hypertonic Na^+^ solution into the cerebral ventricle did not induce aversion to salt in *Na*_*x*_-KO mice, in contrast to wild-type animals [33]. Importantly, the aversion to salt was not induced by the infusion of a hyperosmotic mannitol solution with physiological [Na^+^] in either genotype of mice, suggesting that Na_x_ is involved in the [Na^+^]-specific sensing mechanism in the brain [33].

After water deprivation, *Na*_*x*_-KO mice showed marked neuronal activation in the SFO and OVLT compared with WT mice, as estimated by Fos immunoreactivity [65]. The behavioral phenotype of *Na*_*x*_-KO mice was completely recovered by a site-directed transfer of the *Na*_*x*_ gene with an adenoviral vector into the SFO (Fig. 1c) [33]. These data clearly indicate that the SFO is the primary locus of [Na^+^] sensing in the brain for the control of salt intake behavior and that Na_x_ plays a critical role in the sensing mechanism.

## Molecular properties of Na_x_ in vitro

**Fig. 2:**
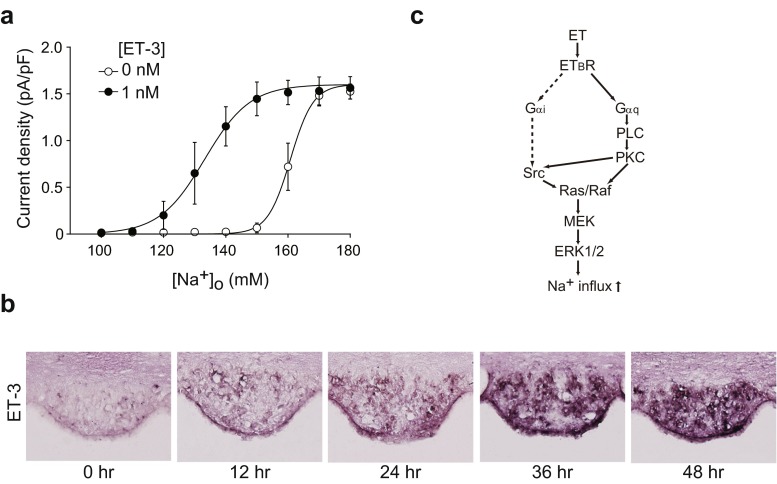
Endothelin-3 (*ET-3*) signaling shifted the [Na^+^]_o_ dependency of Na_x_ activation to the lower side. **a** Relationships between the current density and [Na^+^]_o_ in the presence or absence of 1 nM ET-3; *n* = 6 for each. **b** In situ hybridization for detection of ET-3 expression in the SFO of WT mice. Brains were obtained from mice provided freely with food and water (0 h) or from those provided only with food during the indicated period (*12*, *24*, *36*, and *48 h*). Sections on the same slide are shown. **c** Activation cascades of Na_x_ by ET_B_R signaling. The pathway indicated by *dotted lines* was suggested not to work for the Na_x_ activation. Reproduced with permission from [35]

We speculated that Na_x_ may open in response to changes in extracellular [Na^+^] ([Na^+^]_o_) and function as the [Na^+^] sensor in the brain. We verified this possibility by imaging analysis of changes in the intracellular [Na^+^] ([Na^+^]_i_) when the [Na^+^]_o_ was raised stepwise from the lower amount [34]. When a series of Na^+^ solutions higher than the physiological level were applied to Na_x_-positive cells isolated from the SFO, persistent Na^+^ influx appeared (Fig. 1d) [34]. The threshold value of Na_x_ for [Na^+^]_o_ was ∼150 mM ([19]; see also Fig. 2a). These [Na^+^]-sensitive cells were insensitive to the rise in osmolality or [Cl^−^]_o_ (Fig. 1e) [34]. As expected, no SFO cells derived from *Na*_*x*_-KO mice showed such responses, and transfection of Na_x_ cDNA conferred [Na^+^]_o_ sensitivity on the cells from *Na*_*x*_-KO mice [53]. Notably, the Na_x_-immunoreactive cells in these loci were revealed to be glial fibrillary acidic protein (GFAP)-positive glial cells (astrocytes and ependymal cells) [67]. These data indicate that Na_x_ is a Na channel sensitive to an increase in the [Na^+^]_o_ and that glial cells are sensing cells.

## [Na^+^] dependency of Na_x_ in vivo

As aforementioned, [Na^+^] is strictly controlled at 135–145 mM in the blood and CSF of mammals, including humans [58]. In order to maintain the physiological level strictly, the active range of sensitivity of brain [Na^+^] sensor(s) should be within this range. However, the apparent threshold value of Na_x_ activation was ∼150 mM in vitro, as described above [34]. Therefore, it was presumed that the threshold value of Na_x_ for [Na^+^]_o_ must be modulated in vivo by some unknown mechanism.

Endothelin receptor B (ET_B_R) is predominantly expressed in glial cells in the brain [36] and extremely highly expressed in the SFO [31]. Furthermore, ET peptides and their receptors are intimately involved in the physiological control of systemic blood pressure and Na homeostasis [43]. It was thus tempting to speculate that ET is involved in signaling mechanisms mediated by sensor molecules such as Na_x_ in the SFO.

By our in situ hybridization, *ET*-*3*, but not *ET*-*1* or *ET*-*2* messenger RNA (mRNA), was detected in some cells inside the SFO [35]. Moreover, ET-converting enzymes (Ece1 and Ece2), proteases responsible for the conversion of inactive ET precursors (big endothelin) to bioactive mature forms [41], were also expressed in the SFO in a similar manner [35]. This situation suggested the presence of autocrine or paracrine signaling mechanisms for ET in the SFO (see below).

## Enhancement mechanism of Na_x_ sensitivity in vivo by ET-3

We examined the effects of ET-3 on the [Na^+^]_o_ dependency of Na_x_ by using the patch clamp method [35]. [Na^+^]_o_-sensitive inward currents were observed when the “high Na^+^ solution” ([Na^+^]_o_ = 170 mM) was applied to Na_x_-positive SFO cells derived from WT mice [35]. The dose response curve of the [Na^+^]_o_-dependent response reached a maximum at ∼170 mM without ET-3; *C*_1/2_ was 161 mM (Fig. 2a; [ET-3] = 0 nM). When 1 nM ET-3 was applied, the response curve of [Na^+^]_o_ dependency shifted to the lower side; the *C*_1/2_ value shifted to 133 mM (Fig. 2a; [ET-3] = 1 nM). This condition may reflect a physiological situation in vivo.

During dehydration, *ET*-*3* mRNA levels in the SFO increased in a time-dependent manner (Fig. 2b; approximately ten fold at 36 h compared to 0 h). On the other hand, the expression of ET_B_R was not regulated by dehydration [35]. The activation of Na_x_ via ET_B_R was revealed to be mediated by a PKC pathway that activates ERK1/2 downstream [35] (Fig. 2c). The phosphorylation of ERK1/2 was consistently and markedly enhanced in the SFO tissues of dehydrated mice [35].

Cells in the SFO, which lacks a BBB, may be exposed to circulating hormones, including ETs [61]. However, ET-3 levels in the plasma and CSF remained low even after 1–2 days of dehydration (ranging from 13 to 32 pM) [35]. Because ET-3 at 50 pM did not affect Na_x_ gating, ET-3 locally produced in the SFO probably stimulates Na_x_-positive glial cells through ET_B_R in an autocrine or paracrine fashion.

We further demonstrated that a specific blocker of ET_B_R attenuated the salt-aversive behavior in WT mice induced by dehydration [35]. Even in slightly dehydrated animals, induction of the expression of ET-3 in the SFO may lead to a stimulation of ET_B_R signaling and a significant enhancement of the sensitivity of Na_x_ to [Na^+^]_o_, which may help animals respond to dehydration robustly.

## Direct interaction between Na_x_ channels and α subunits of Na^+^/K^+^-ATPase

**Fig. 3:**
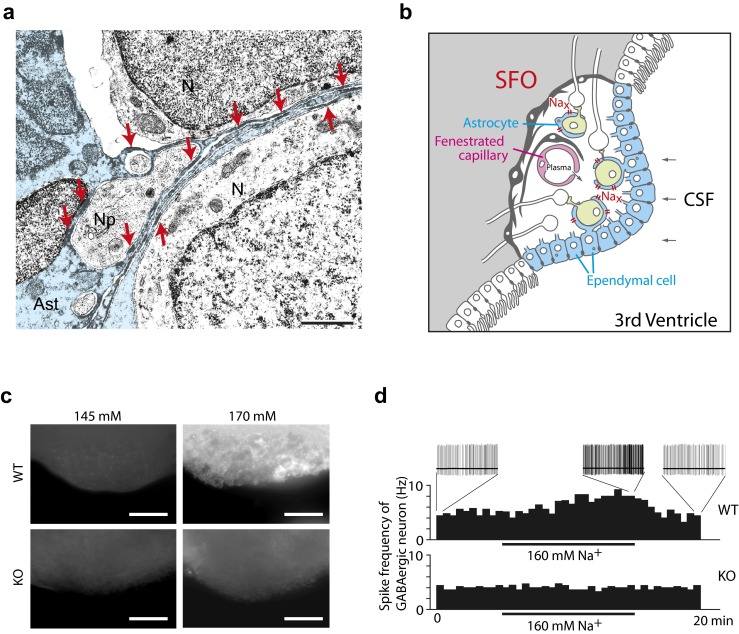
Na_x_ channels control lactate signaling from glial cells to neurons for [Na^+^] sensing in the SFO. **a** Immunoelectron microscopy of the SFO using anti-Na_x_ antibody. Neurons (*N*) and their processes (*Np*) are enveloped with the immunopositive thin processes of an astrocyte (*Ast*; *blue*). *Red arrows* point to immunopositive signals. Neurons and their processes, including synapses, are surrounded by immunopositive thin processes of astrocytes. *Scale bars*, 1 μm. **b** Schematic drawing of Na_x_-positive ependymal cells and astrocytes in the SFO. The SFO is characterized by the presence of neuronal cell bodies and extensive networks of fenestrated capillaries that allow components of the plasma to leak into the intercellular space. The SFO has contact with the CSF through a single layer of Na_x_-positive ependymal cells. **c** Imaging analyses of the uptake of glucose in the SFO using a fluorescent glucose derivative. The SFO tissues isolated from wild-type (*WT*) and *Na*_*x*_-KO (*KO*) mice were incubated with the fluorescent glucose analog in 145 mM (*left column*) or 170 mM (*right column*) Na^+^ solution. *Scale bars*, 50 μm. **d** Control of spike frequency of GABAergic neurons in the SFO by Na^+^. The SFO tissues from WT and *Na*_*x*_-KO mice were treated with the high-Na^+^ solution. Na_x_ is indispensable for [Na^+^]-dependent potentiation of the GABAergic firing in the SFO. Reproduced with permission from [67] (**a**), [52] (**b**), and [60] (**c**, **d**)

Our analyses using electron microscopy revealed that Na_x_ channels are specifically expressed in perineuronal processes of astrocytes and ependymal cells enveloping particular neural populations, including GABAergic neurons in the SFO (Fig. 3a). These Na_x_-positive glial cells were sensitive to an increase in the [Na^+^]_o_ [67], indicating that glial cells, not neurons, are the primary site of [Na^+^] sensing (Fig. 3b). These findings imply that there exists a signal transfer from Na_x_-positive glial cells to SFO neurons because neuronal activity in the SFO is involved in the control of body fluid homeostasis. However, there was no clue as to the signaling substance or mechanism at that time.

We therefore started with a screening for molecules interacting with the cytoplasmic domains of Na_x_ to better understand the physiological processes involving Na_x_ in glial cells. This screening revealed that Na_x_ channels stably interact with α1 and α2 subunits of Na^+^/K^+^-ATPase via its carboxyl-terminus region [60]. Subsequent detailed analyses revealed a close physical and functional coupling between Na_x_ and Na^+^/K^+^-ATPase: Binding of Na_x_ to Na^+^/K^+^-ATPase is requisite to [Na^+^]_i_-dependent activation of Na^+^/K^+^-ATPase, and [Na^+^]_o_-dependent activation of Na_x_ leads to stimulation of Na^+^/K^+^-ATPase activity [60].

## Na^+^-dependent metabolic enhancement of the Na_x_-positive glial cells

Activation of Na^+^/K^+^-ATPase potentially stimulates anaerobic metabolism of glucose in glial cells and produces lactate as the end product. To examine whether the Na_x_ channel is indeed involved in the energy control system in the Na_x_-positive glial cells in vivo, we performed an imaging analysis of the uptake of glucose in the SFO using a fluorescent glucose derivative. [Na^+^]_o_-sensitive glucose uptake was obviously detected selectively in the WT but not in the *Na*_*x*_-KO tissues: After incubation with a hypertonic Na^+^ solution, an intensively labeled mesh-like structure became apparent in the SFO obtained from WT mice [60] (Fig. 3c). This result suggested that fine glial processes in the SFO actively took up the fluorescent derivative of glucose. The enhancement of glucose uptake was completely abolished by an Na^+^/K^+^-ATPase inhibitor, ouabain, indicating that the activity of Na^+^/K^+^-ATPase plays an essential role in the glucose demand induced by the elevation of the [Na^+^]_o_ [60]. Consistently, lactate release from the SFO tissue of WT, but not of *Na*_*x*_-KO mice, was upregulated by incubation with hypertonic Na^+^ solution [60].

## Lactate signaling from glial cells to neurons

We next examined the possibility that lactate mediates the signal transfer from glial cells to neurons to control activity. In the SFO, GABAergic neurons are one of the major neuronal types surrounded by Na_x_-positive glial processes [67]. In electrophysiological experiments using tissue slices, we found that the GABAergic neurons in the SFO are spontaneously firing, and the firing frequency in the slices of WT mice gradually increased on application of hypertonic Na^+^ (Fig. 3d, WT). In contrast, the activity of GABAergic neurons in the SFO of *Na*_*x*_-KO mice was not potentiated by hypertonic Na^+^ (Fig. 3d, KO). GABAergic neurons of both genotypes were increased when lactate was directly added at 1 mM to the perfusate [60]. An inhibitor of monocarboxylate transporters (MCTs), which transport lactate across the membrane, inhibited the Na^+^-dependent potentiation of the GABAergic firing [60]. Subsequent analyses revealed that the underlying mechanism of the activation was depolarization of GABAergic neurons, due, in part, to the inactivation of the ATP-sensitive K channel (Kir6.2/K_ATP_ channel): the K_ATP_ channel closes in response to an increase of intracellular ATP level as a result of lactate metabolism in neurons [60]. These data clearly indicate that lactate released from glial cells serves as an energy substrate to upregulate the firing activity of the GABAergic neurons. This lactate signaling appears to play a crucial role in the control of neuronal activities involved in the Na intake behavior in the brain.

## Summary of the [Na^+^]-sensing mechanism in the SFO for the control of salt intake behavior

**Fig. 4:**
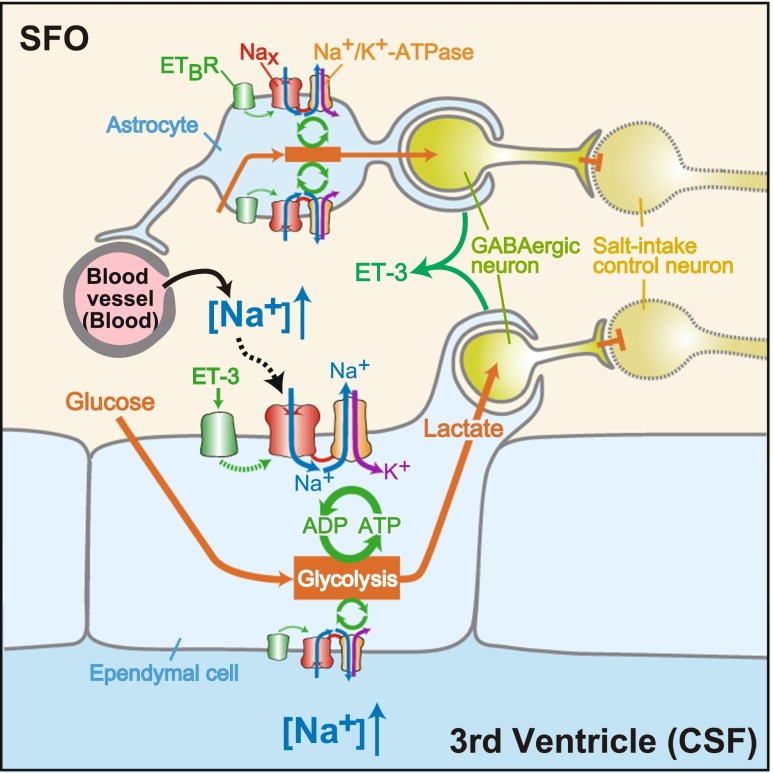
Overview of the [Na^+^]-sensing mechanism and Na_x_-dependent regulation of neuronal activity in the SFO. Reproduced with permission from [55]

Based on our findings, a schematic overview of the cellular mechanisms for [Na^+^] sensing and [Na^+^]-dependent regulation of neural activities in the SFO is presented in Fig. 4. The sensory CVOs, including the SFO, are characterized by the extensive networks of fenestrated capillaries which allow ingredients of plasma to be released to the intercellular space. Their ventricular side is partitioned by an ependymal cell layer facing the third ventricle. Na_x_ channels populate perineural processes of astrocytes and ependymal cells in the SFO. Even under hydrated (normal) conditions, ET-3 level expressed in the SFO could modulate the [Na^+^]_o_ dependency of Na_x_ and make Na_x_ sensitive to an increase in [Na^+^]_o_ in the physiological range. When animals are dehydrated, [Na^+^] in plasma and CSF significantly increases above the usual level. Under such conditions, the [Na^+^]_o_ exceeds the threshold of Na_x_, Na_x_ channels open, and the [Na^+^]_i_ is increased. This leads to activation of Na^+^/K^+^-ATPase in these cells. Activated Na^+^/K^+^-ATPase consumes ATP higher than the usual level to pump out Na^+^. To fuel Na^+^/K^+^-ATPase with ATP, the glial cells enhance glucose uptake to stimulate anaerobic glycolysis. Lactate, the end product of the anaerobic glycolysis, is released from the glial cells and supplied to neurons, including GABAergic neurons, through the processes enveloping them. Lactate stimulates the activity of the GABAergic neurons through production of ATP, which presumably leads to the regulation of hypothetic neurons involved in the control of salt intake behavior. In dehydrated KO mice, the [Na^+^]-dependent stimulation of glycolysis is impaired and the activity of the GABAergic neurons is not promoted.

## Essential hypernatremia caused by autoimmunity to Na_x_

“Essential hypernatremia” is clinically characterized by chronic elevation of plasma [Na^+^] with an inappropriate lack of thirst and upward resetting of the osmotic set point for vasopressin release, thereby resulting in persistent hypernatremia with a euvolemic state [6, 18, 68]. In most cases of essential hypernatremia, structural abnormalities are commonly detected in the hypothalamic-pituitary area, as a result of trauma, tumors, or inflammation. However, several cases of essential hypernatremia without demonstrable hypothalamic structural lesions have been reported, although the precise mechanism(s) has(have) not yet been elucidated [8, 14, 15, 20, 29, 59].

We examined a patient with essential hypernatremia associated with abnormal reductions in water intake and vasopressin release without demonstrable hypothalamic structural lesions and found that she developed autoantibodies to Na_x_ [32, 54]. Passive transfer of the immunoglobulin (Ig) fraction of the patient’s serum in WT mice reproduced her symptoms. This was revealed to be induced by complement-mediated cell death in the CVOs, where Na_x_ is specifically expressed [32]. The SFO and OVLT have projections to the SON and PVN, which are responsible for regulating the production/release of vasopressin (Fig. 1a) [46, 64]. Histological damage to the SFO and OVLT may be the reason for the dysregulation in vasopressin production/release. This defect in the regulation of vasopressin appears to have caused serious symptoms in the patient.

Since sensory CVOs lack a BBB, antibodies easily leak from blood vessels into these loci [11]. This indicates that cell surface proteins, including channels and transporters in the sensory CVOs, may be easy targets of autoantibodies. Neurons or glial cells in the sensory CVOs are known to express receptors for multiple circulating peptides, including angiotensin II and natriuretic peptides [49]. These receptors may also be potential targets for autoantibodies. We very recently identified more patients with essential hypernatremia whose sera contained autoantibodies that were specifically reactive to the SFO (in preparation). Thus, examination of autoantibodies reactive to the sensory CVOs represents an option for clinical tests of patients with chronic body-fluid disorders.

## Na_x_ expression in the other loci in the brain and peripheral tissues

Beside the SFO and OVLT, clusters of *lacZ* expression in the *Na*_*x*_-KO mice were also observed in some specific loci in the brain: the medial preoptic area, the anterior and dorsomedial part of the hypothalamic area, dorsomedial part of the interpeduncular nucleus, medial part of the median raphe (so-called rhabdoid nucleus), mesencephalic nucleus of the fifth cranial nerve, medial habenular nucleus, median eminence, and neurohypophysis [65]. In addition, relatively weak *lacZ* expression was detected in the cerebral cortex in layer IV of the lateral area (from the most anterior portion to the end of the posterior portion of the cortex) and the basolateral amygdala [65]. This expression was confirmed by immunohistochemistry using our antibody (our unpublished data).

Grob et al. reported that neurons in the median preoptic nucleus (MnPO) in rats responded to a change in the extracellular sodium concentration [27]. MnPO is a midline structure situated on the anterior wall of the third ventricle between the SFO and OVLT and receives neural connections from these two loci [49]. They also showed that the MnPO in rats was positive for Na_x_ expression by in situ hybridization. As aforementioned, we detected X-gal staining in the medial preoptic area but not in the MnPO in *Na*_*x*_-KO mice [65]. Recently, the same group reported that this was attributed to the species difference by showing that Na_x_ expression in the MnPO is observed in rats but not in mice [50]. However, as far as we examined with our antibody, of which specificity was confirmed by immunostaining using *Na*_*x*_-KO mice [34], we could not detect any signals in rat MnPO immunohistochemically (our unpublished data). Because MnPO has a BBB, it may not primarily participate in the [Na^+^] sensing in the brain, though it should be an important locus for integration of the neural information for body fluid homeostasis.

In the peripheral nervous systems, Na_x_ expression was detected in the dorsal root ganglia and non-myelinating Schwann cells [65, 66]. In previous papers by other groups, relatively high levels of *Na*_*x*_ mRNA were detected outside the nervous system, particularly in the lung, heart, and perinatal uterine smooth muscle by Northern blot analysis [2, 23], and in the kidney by reverse transcription-polymerase chain reaction (RT-PCR) [2]. We examined the localization of Na_x_ throughout the visceral organs at the cellular level [66]: In the lung especially of neonates, robust Na_x_ signals were observed in the alveolar type II cells, which actively absorb sodium and water to aid gas exchange through the alveolar surface [47]. The myometrium of the pregnant uterus was significantly positive for Na_x_ expression [65]. In visceral organs including lung, heart, intestine, bladder, kidney and tongue, a subset of Schwann cells within the peripheral nerve trunks and ganglia were highly positive for Na_x_. Further studies revealed that these Na_x_-positive cells were non-myelinating Schwann cells of sympathetic and/or parasympathetic nerve fibers surrounding blood vessels [66]. Recently, Lara et al. reported that Na_x_ is expressed in the thick ascending limb and collecting duct cells in rat kidney [44]. However, as far as we examined *lacZ* expression in the *Na*_*x*_-KO mice and Na_x_ expression in wild-type mice by immunohistochemistry, we could not verify the expression in the kidney (our unpublished data).

## Future directions

In this review, we summarized a series of our studies on the [Na^+^]-sensing mechanism in the brain for the control of salt intake behavior. Although some candidates for osmosensors have been postulated, the mechanism for osmosensing is still controversial [42, 55], in contrast to the [Na^+^] sensing. A [Na^+^]-sensing mechanism in the brain is also considered to be involved in the control of blood pressure. Excess dietary salt is one of the decisive factors for the rise of blood pressure [40]. Slightly elevated plasma [Na^+^] with apparent normovolemia is often observed in hypertensive humans [10]. Increases in [Na^+^] in CSF by central infusion of Na^+^-rich artificial CSF cause sympathetic hyperactivity and hypertension [45]. Therefore, some sensing mechanism for [Na^+^] in CSF is considered to be a decisive factor in salt-sensitive hypertension, though the precise mechanism has not been elucidated [10, 56]. It is noteworthy that lesions of the anteroventral third ventricle region (AV3V), which encompasses the SFO and OVLT, prevent the development or reverse hypertension in several hypertensive rats including Dahl salt-sensitive and DOCA-salt rats [9, 12, 13, 19, 26, 30]. In addition, it was reported that the ventral lamina terminalis mediates enhanced cardiovascular responses of rostral ventrolateral medulla neurons during increased dietary salt [1]. It would be important to examine whether the [Na^+^] sensing by Na_x_ in the SFO and/or OVLT is involved in the salt-sensitive control of blood pressure.
